# Contribution of collagen XIII to lung function and development of pulmonary fibrosis

**DOI:** 10.1136/bmjresp-2023-001850

**Published:** 2023-12-12

**Authors:** Oula Norman, Jarkko Koivunen, Riitta Kaarteenaho, Antti M Salo, Joni M Mäki, Johanna Myllyharju, Taina Pihlajaniemi, Anne Heikkinen

**Affiliations:** 1 ECM-Hypoxia Research Unit, Faculty of Biochemistry and Molecular Medicine, University of Oulu, Oulu, Finland; 2 Research Unit of Biomedicine and Internal Medicine and Medical Research Center Oulu, University of Oulu, Oulu, Finland; 3 Center for Internal Medicine and Respiratory Medicine, Oulu University Hospital, Oulu, Finland

**Keywords:** Respiratory Function Test, Respiratory Muscles, Interstitial Fibrosis

## Abstract

**Background:**

Collagen XIII is a transmembrane collagen associated with neuromuscular junction development, and in humans its deficiency results in congenital myasthenic syndrome type 19 (CMS19), which leads to breathing difficulties. CMS19 patients usually have restricted lung capacity and one patient developed chronic lung disease. In single-cell RNA sequencing studies, collagen XIII has been identified as a marker for pulmonary lipofibroblasts, which have been implicated in the resolution of pulmonary fibrosis.

**Methods:**

We investigated the location and function of collagen XIII in the lung to understand the origin of pulmonary symptoms in human CMS19 patients. Additionally, we performed immunostainings on idiopathic pulmonary fibrosis (IPF) samples (N=5) and both normal and fibrotic mouse lung. To study whether the lack of collagen XIII predisposes to restrictive lung disease, we exposed *Col13a1*-modified mice to bleomycin-induced pulmonary fibrosis.

**Results:**

Apparently normal alveolar septum sections of IPF patients′ lungs stained faintly for collagen XIII, and its expression was pinpointed to the septal fibroblasts in the mouse lung. Lung capacity was increased in mice lacking collagen XIII by over 10%. In IPF samples, collagen XIII was expressed by basal epithelial cells, hyperplastic alveolar epithelial cells and stromal cells in fibrotic areas, but the development of pulmonary fibrosis was unaffected in collagen XIII-deficient mice.

**Conclusions:**

Changes in mouse lung function appear to represent a myasthenic manifestation of collagen XIII deficiency. We suggest that respiratory muscle myasthenia is the primary cause of the breathing problems suffered by CMS19 patients in addition to skeletal deformities. Induction of collagen XIII expression in the IPF patients′ lungs warrants further studies to reveal collagen XIII-dependent disease mechanisms.

**WHAT IS ALREADY KNOWN ON THIS TOPIC**

- Collagen XIII is relatively highly expressed in the lung by alveolar fibroblasts and human congenital myasthenic syndrome type 19 (CMS19) patients lacking collagen XIII suffer from breathing difficulties in conjunction with myasthenia.

**WHAT THIS STUDY ADDS**

- We demonstrate that collagen XIII is associated with alveolar basement membranes next to the alveolar fibroblasts. Mice lacking collagen XIII exhibit increased vital capacity and chest extendibility because of the myasthenic phenotype. Human idiopathic pulmonary fibrosis patients show increased collagen XIII expression in disease-specific cell types such as hyperplastic epithelial cells, but in mice the lack of collagen XIII does not affect the development of pulmonary fibrosis.

**HOW THIS STUDY MIGHT AFFECT RESEARCH, PRACTICE OR POLICY**

- The results are of interest in trying to understand the role of collagen XIII-positive lung fibroblasts in health and disease. In the CMS19 disease model, the lack of collagen XIII does not seem to affect lung development or structure, and thus efforts should remain focused on the treatment of the myasthenia.

## Introduction

Collagen XIII is a transmembrane collagen associated with neuromuscular junction development, osteal integrity and pulmonary fibroblasts.^1–3^ In humans its deficiency results in congenital myasthenic syndrome type 19 (CMS19), which manifests as muscle weakness, leading to swallowing and breathing difficulties, the latter of which may progress fatally.^4^ Most CMS19 patients experience respiratory crises either spontaneously or in conjunction with respiratory tract infections at an early age, often requiring mechanical ventilation. Even in adulthood, many patients have had severely decreased vital capacity together with spinal and thoracic deformities. At least one individual with CMS19 developed chronic lung disease.^4–9^

Collagen XIII is expressed in the lung at a relatively high level,^10 11^ but its function is still unclear. Recently, however, it has been identified in several single-cell RNA sequencing (scRNA-seq) studies as accurately delineating a pulmonary lipofibroblast population localised in the alveolar interstitium.^3 12 13^ These collagen XIII-expressing lipofibroblasts contain intracellular lipid droplets and express perilipin 2.^14^ It has been suggested that this cell population may mediate endothelial and epithelial function via angiopoietin, Met and Slit signalling.^15^ Another function of lipofibroblasts is reported to be the uptake of fatty acids and the synthesis and transfer of neutral lipids to alveolar type II cells for surfactant production.^16 17^ Collagen XIII binds *in vitro* to several extracellular matrix components such as perlecan, nidogen 2, fibronectin and colQ.^18 19^ Moreover, the shed ectodomain of collagen XIII inhibits fibronectin network assembly, and cell adhesion on vitronectin.^20 21^ Of the collagen-binding integrins, collagen XIII interacts with α1β1 and α11β1.^22 23^

Idiopathic pulmonary fibrosis (IPF), the most common form of pulmonary fibrosis, affects approximately 3 million people worldwide, with a median survival of around 2.5–5 years after diagnosis.^24–26^ It is characterised by a typical pattern of histological changes called usual interstitial pneumonia (UIP). UIP consists of cystic airspaces with thickened walls called honeycombing, dilatation of the bronchi, thickening of the peripheral alveolar septa, formation of fibroblastic foci and hyperplastic alveolar epithelial cells lining damaged alveoli.^26^ UIP typically affects the basal and peripheral parts of the lung most severely. The cuboidal hyperplastic epithelial cells that line damaged alveoli, in a phenomenon sometimes called bronchiolisation, are thought to represent a regenerative process originating from stem cells, and have been implicated as having profibrotic properties.^27 28^

In the pathogenesis of pulmonary fibrosis, alveolar fibroblasts, including lipofibroblasts, have been reported to give rise to pathological myofibroblasts^29^ and collagen-producing fibroblasts.^12^ Conversely, transdifferentiation of pathological myofibroblasts to lipofibroblasts is reportedly beneficial for the resolution of bleomycin-induced fibrosis in mice.^29 30^ In additional scRNA-seq studies on IPF, collagen XIII expression has been assigned to a recently identified epithelial cell population, disease-enriched aberrant basaloid cells, coexpressing markers of the basal epithelium, mesenchyme, senescence, development and repair, and it has been suggested that these may exhibit progenitor-cell properties.^28^

We aimed to identify the location and function of collagen XIII in the lung in order to understand the origin of the pulmonary symptoms observed in CMS19 patients and to determine whether collagen XIII is involved in the pathogenesis of pulmonary fibrosis. To achieve this, we used collagen XIII knockout mice,^10^ which have been shown to recapitulate the main aspects of the human CMS19 phenotype,^10 19^ including muscle weakness, skeletal abnormalities and shallower breathing than in their wild-type littermates.^2 19^ We complemented our studies by staining IPF patient samples for immunohistochemical expression of collagen XIII.

We show here collagen XIII expression in both the human and mouse lung and describe its induction in certain cell types in IPF. Measurements of mouse lung function indicate collagen XIII-dependent changes in knockout mice and suggest myasthenia as the primary cause behind breathing problems in addition to skeletal deformities in CMS19 patients. The development of lung fibrosis is unaffected in mice lacking collagen XIII.

## Methods

### Human tissue samples

The human samples used here consisted of diagnostic surgical lung biopsies obtained from five IPF patients at Oulu University Hospital in 1994–2015. IPF was diagnosed in accordance with the international guidelines. The biopsy samples were fixed with formalin, embedded in paraffin and sectioned for staining.

### Mice

The generation of *Col13a1* knockout (*Col13a1^−/−^*) mice (B6.129-Col13a1^tm3.1Pih^/Oulu; EM:09878) is described in detail in Latvanlehto *et al.*^10^ The generation of *Col13a1^tm/tm^* mice (B6JOlaHsd.129S6-Col13a1^tm4.1Pih^/Oulu; EM09323) is described in Härönen *et al*.^19^ Briefly, the furin cleavage site of collagen XIII was mutated from -RRRR- to -ATAA- to prevent cleavage of the membrane-bound collagen XIII. Wild-type littermates were used as controls. Both sexes were used in all the experiments. The experimental unit was a single animal. A subset of the mice used here were also used in another publication on suitable quantitative PCR reference genes.^31^ More details are presented in online supplemental methods. The total number of mice was 233.

10.1136/bmjresp-2023-001850.supp6 Supplementary data

### Bleomycin mouse model for pulmonary fibrosis

At the age of 6 weeks, the mice were transferred from the specific pathogen free facility to the research facilities and the diet was supplemented with banana and hazelnut cocoa spread to minimise malnutrition and mortality after the bleomycin administration. At 8 weeks, the mice were anaesthetised with isoflurane and given 1.25 U/kg bleomycin (Baxter) diluted with sterile isotonic saline to a volume of 1.67 µL/g of body weight by oropharyngeal aspiration, as described by Barbayianni *et al*.^32^ After 4 weeks of follow-up, lung function measurements were taken and the tissues were collected for analysis. During the follow-up period, the mice were weighed and assessed daily. Humane endpoints were defined as weight loss of over 20% or any kind of moderate or severe distress to the mouse. No exclusion criteria were used apart from lung function measurements. During these steps, the researchers were blind to genotype identity.

The left lung (and optionally the right middle lobe when used for electron microscopy) was subjected to intratracheal fixation with 4% paraformaldehyde (PFA) at a pressure of 25 cmH_2_O for 2 min to open the airways, with right bronchi ligated to prevent fixation. The right lung lobes were dissected, snap frozen in liquid nitrogen and stored at −80°C. The left lobe was immersed in 4% PFA for 24 hours and embedded in paraffin.

The right middle lobe was cut into small pieces and immersed with 1% glutaraldehyde, 4% PFA/0.1 M phosphate buffer, pH 7.3, overnight at +4 °C, postfixed with 1% osmium tetroxide, dehydrated in acetone and embedded in Epon LX112. Thin sections were analysed with a Tecnai G2 Spirit 120 kV transmission electron microscope (TEM) and imaged with a Quemesa CCD camera.

### Immunohistochemistry

The human lung slides were baked at 55°C for 1 hour, deparaffinised with xylene and rehydrated through descending concentrations of ethanol. Epitope retrieval was performed by boiling the slides in 10 mM Tris- 1 mM EDTA, pH 9, for 15 min. After washing with 0.1% Tween-20 in phosphate-buffered saline (PBST), peroxidase blocking solution (Dako S2023) was applied for 5 min and the slides washed again. They were then incubated with anti-collagen XIII antibody (Atlas Antibodies Cat# HPA050392) diluted 1:300 in blocking buffer containing 1% bovine serum albumin and 22.52 mg/mL glycine in PBST. After washing, the slides were incubated with Envision polymer (Dako K5007) for 30 min, followed by further washes. They were then incubated with DAB working solution (Dako K5007) for 3 min, washed with water, counterstained with haematoxylin and rinsed in 0.75% ammonium water before dehydration and mounting.

### Immunofluorescence and correlative light and electron microscopy

Epitope retrieval from paraffin-embedded 5 µm sections of lung tissue was performed by incubating the slides in DIVA decloaker solution (Biocare Medical) for 40 min at 100°C, followed by Hot Rinse solution (Biocare Medical) for 5 min and phosphate-buffered saline (PBS) for 5 min. The sections were incubated with serum-free protein block (Dako) for 5 min and then with anti-collagen XIII antibody (Atlas Antibodies Cat# HPA050392) diluted 1:100 in PBS overnight. After three washes with PBS, the sections were incubated with Cy3 Goat Anti-Rabbit IgG (Jackson ImmunoResearch Labs Cat# 111-165-144) diluted 1:300 in PBS for 30 min and again washed three times with PBS. The coverslips were mounted with Immu-Mount (Thermo Scientific). The samples were imaged with a Leica SP8 Falcon confocal microscope with only individual optical sections used. After imaging, the coverslip was detached by incubating in PBS. The sections were postfixed with 1% osmium tetroxide, dehydrated in acetone and embedded in Epon LX112 using gelatin capsules. The region of interest was identified microscopically, trimmed and thin sections were prepared. The samples were imaged with a Tecnai G2 Spirit electron microscope. The light and electron microscopy images were correlated using nuclear staining with the eC-CLEM plugin^33^ for Icy.^34^

### Lung function measurements

Mice aged 3 and 6 months old were anaesthetised with a subcutaneous injection of 0.4 mg/kg fentanyl, 10 mg/kg midazolam and 1.5 mg/kg medetomidine. The trachea was cannulated with an 18 G cannula from a midline incision after local anaesthesia with 0.1% lidocaine. The cannula was connected to a flexiVent FX Module 2 ventilator (SCIREQ, Montreal) equipped with a Forced Expiration Extension add-on. The mice were ventilated at 150 breaths per minute with a tidal volume of 10 mL/kg body weight and a positive end-expiratory pressure of 3 cmH_2_O. Muscle relaxation was achieved with an intraperitoneal injection of 4 mg/kg body weight rocuronium bromide (B. Braun). Lung function was measured by running a Deep Inflation manoeuvre, a single frequency FOT (‘Snapshot-150’), a QuickPrime-3 manoeuvre, a stepwise pressure-volume loop (‘PVs-P’) and a negative pressure forced expiration (NPFE’) manoeuvre with approximately 5 s intervals of regular ventilation between these measurements. Triplicate measurements were made on each mouse and averaged for the final values. Measurements with software-calculated coefficient of determination values below 0.95 for any manoeuvre and measurements that showed signs of airway instability, such as spontaneous breathing efforts, cannula obstruction or leaks, were excluded from the analysis. The assessment of measurement suitability was done blind to genotype identity. More details are presented in online supplemental methods.

### Histological scoring

The left lungs were embedded in paraffin in the frontal plane and sectioned to 5 µm sections at 200 µm intervals. The sections were stained with Masson’s trichrome. One section immediately before and one after the main bronchus were used for histological analysis. The slides were scanned at ×40 magnification with a NanoZoomer S60 slide scanner (Hamamatsu). Regions of interest with only lung tissue in them were drawn with QuPath^35^ and the images downsampled by a factor of five with a script^36^ to enable processing with ImageJ. 10 fields of size 0.9×0.9 mm were randomly sampled using an ImageJ script modified from Mason^37^ for each section and graded using the modified Ashcroft scale^38^ independently by three observers blinded to the sample identities. The scores from all observers for a given field were averaged and then the scores for all the fields in the sample were averaged to obtain a score for the entire sample.

### 4-Hydroxyproline assay

The inferior lobe of the right lung was lyophilised and hydrolyzed in 6M HCl at 120°C overnight, and the collagen content determined by a colorimetric method for 4-hydroxyproline described by Berg.^39^ The result was calculated as total 4-hydroxyproline in the lobe.

### Reverse transcription quantitative real-time PCR (RT-qPCR)

RT-qPCR was performed as described previously.^31^ The primer sequences for *Col13a1* were forward 5’- GAAGCCCCGAAGATGTCTCC and reverse 5’-TGGGSGTCCAGGTCTTCCAG, efficiency 98.9% and standard curve R^2^ 0.991. For *Plin2*, forward 5’- GATTGAATTCGCCAGGAAGA and reverse 5’- TGGCATGTAGTCTGGAGCTG, efficiency 95.6% and standard curve R^2^ 0.997. The annealing temperature was 60°C for all the reactions.

### Statistical analyses

Sample numbers for the bleomycin experiments were calculated *a priori* to obtain an 80% statistical power for detecting 20% changes in lung volume with alpha=0.05, beta=0.2 and an SD of 15% of the control group mean, which resulted in n=9 per group. A one-way analysis of variance (ANOVA) with Tukey’s post hoc test was used to compare means. The normality of the data was assessed from histograms. SPSS Statistics V.26 (IBM) and GraphPad Prism (GraphPad Software) were used for the analyses. A p<0.05 was considered statistically significant.

### Patient and public involvement

Patients were not involved in the research.

## Results

### Collagen XIII expression along alveolar septa in the normal-appearing human lung in IPF

**Figure 1:**
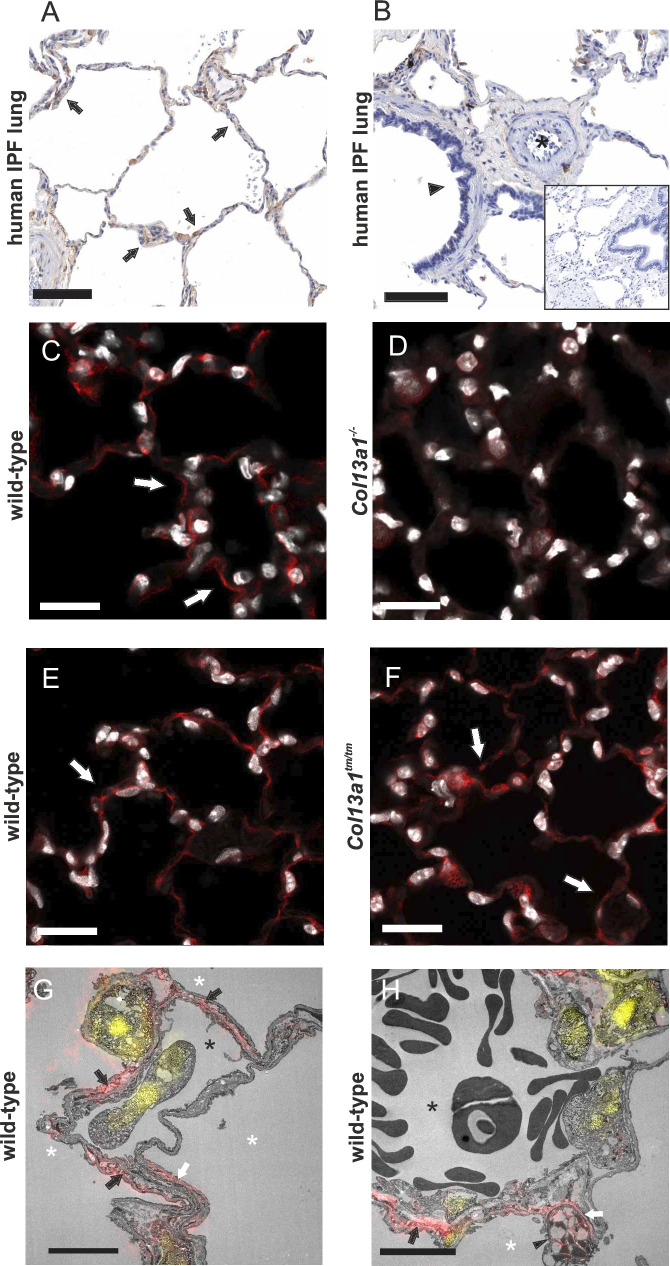
Collagen XIII expression in normal-appearing lung in IPF. Collagen XIII staining (arrows) of (A, B) seemingly normal areas of IPF lung (brown) with a primary antibody-omitted negative control inset in (B), (C) wild-type littermate mouse lung (red), (D) *Col13a1*^*−/−*^ mouse lung, (E) wild-type littermate mouse lung and (F) *Col13a1*^*tm/tm*^ mouse lung. Nuclei are counterstained with DAPI (white). Asterisk: arteriole, arrow head: bronchiole. Scale bars 100 µm (A, B) and 20 µm (C–F). (G, H) CLEM for collagen XIII (red) in wild-type mouse lung. Nuclei (DAPI) in yellow. White asterisks: alveolus. Black asterisk: capillary (G)/venule (H) lumen. White arrow: alveolar basement membrane. Black arrows: regions of the alveolar wall with alveolar fibroblast processes. Arrowhead: type II alveolar epithelial cell. Scale bars 5 µm. White balance and/or brightness have been adjusted for easier viewing. CLEM, correlative light and electron microscopy; IPF, idiopathic pulmonary fibrosis.

To learn about the expression of collagen XIII in the lung, pulmonary sections from five IPF patients were immunostained. In the nearly normal-looking tissue areas outside the fibrotic lesions collagen XIII staining was observed as thin lines along alveolar septa (figure 1A). The vascular structures were generally negative for the collagen XIII signal (figure 1B), and bronchioles in areas with fewer fibrotic lesions were likewise negative for collagen XIII (figure 1B). In the immunohistochemistry images published in the Human Protein Atlas resource (https://www.proteinatlas.org/ENSG00000197467-COL13A1/tissue/lung)^40^, collagen XIII expression is seen in the alveolar septa in non-fibrotic lungs whereas vasculature and bronchioles are negative for collagen XIII. Notably, we used the very same antibody as the Human Protein Atlas in our stainings of IPF lungs.

### Collagen XIII is expressed in mouse alveolar fibroblasts

To further validate and compare the mouse and human expression patterns, we immunostained normal mouse lungs for collagen XIII using collagen XIII knockout (*Col13a1*^−/−^) mice as negative controls to validate the antibody specificity. The staining pattern of collagen XIII in the mouse lungs closely resembled those found in apparently histologically normal regions of human patient samples, with expression in short, continuous wavy lines along the alveolar septa (figure 1C). The knock-out samples were devoid of any signal demonstrating antibody specificity (figure 1D). We also performed the corresponding staining on *Col13a1^tm/tm^* mice, in which the cleavage of collagen XIII is prevented, so that only transmembrane collagen XIII is expressed. The staining pattern of the *Col13a1^tm/tm^* lung for collagen XIII resembles that of the wild-type mice, but the septal lines of the immunosignal are discontinuous and more punctuated in appearance, indicating that the portion of collagen XIII associated with basement membranes is in the shed form and thus lacking in *Col13a1^tm/tm^* mice (figure 1E,F). For a more detailed view, we performed correlative light and electron microscopy on samples of wild-type mouse lung. At the ultrastructural level, collagen XIII staining colocalised with alveolar fibroblasts in association with the basement membranes underlining both type I and type II cells (figure 1G,H).

### Lung histology and fine structure is unaffected in collagen XIII-modified mice

To evaluate whether there were visible differences in lung structure, the histology of both *Col13a1*^−/−^ and *Col13a1*^*tm/tm*^ mice at 3 and 6 months of age was assessed with Masson’s trichrome stainings and compared with littermate wild-type controls in groups of at least five animals of both sexes. No clear differences were observed in the structure of the bronchus or bronchiole, blood vessels or the appearance of alveoli in the lungs of mice with altered collagen XIII expression (online supplemental figure 1).

10.1136/bmjresp-2023-001850.supp1 Supplementary data

To determine whether the total absence of collagen XIII or absence of the shed form led to changes in the integrity of the fine structure, we studied the lung ultrastructure of wild-type and *Col13a1*^−/−^ mice aged 3 months and 6 months using TEM with 4–5 animals/group, including both sexes. No differences were observed in lung structure, cell morphology or the appearance of the lipid droplets, type II cells or the extracellular matrix (online supplemental figure 2). A few *Col13a1*^*tm/tm*^ mice were also studied at the age of 6 months, but differences could not be observed (not shown).

10.1136/bmjresp-2023-001850.supp2 Supplementary data

### Lack of collagen XIII results in increased lung volume

**Figure 2:**
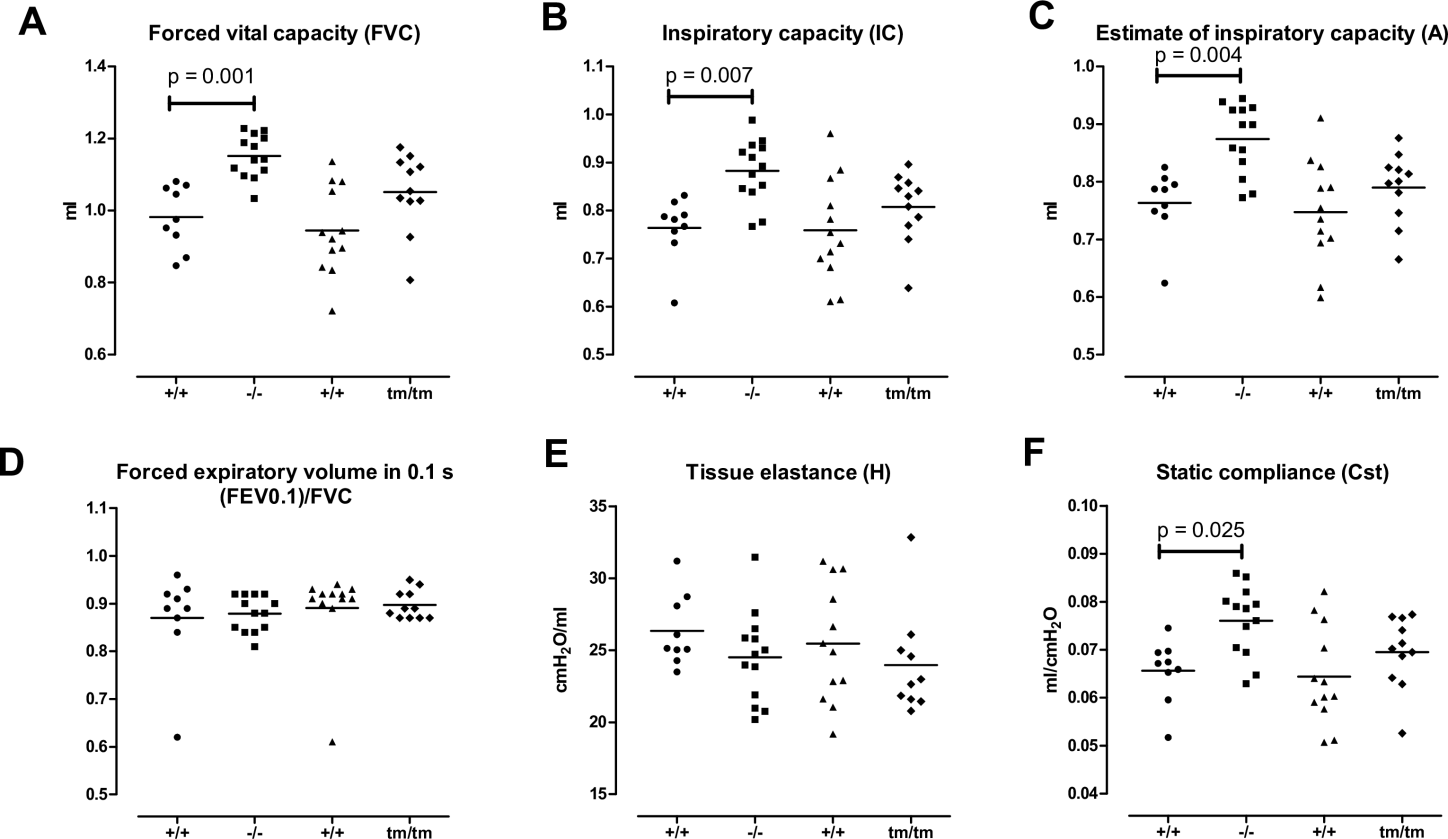
Lung function of *Col13a1* gene-modified mice at 3 months. flexiVent measurements of (A) FVC, (B) IC, (C) A, (D) FEV0.1/FVC, (E) H and (F) Cst. +/+=wild type mice (first column *Col13a1*^*−/−*^ littermate controls N=9, third column *Col13a1*^*tm/tm*^ littermate controls N=12), −/−=*Col13a1*^*−/−*^ mice (N=13), tm/tm=*Col13a1*^*tm/tm*^ mice (N=10–11). Lines at mean. One-way ANOVA with Tukey’s post hoc test. ANOVA, analysis of variance; FEV0.1, forced expiratory volume in 0.1 s; FVC, forced vital capacity; IC, inspiratory capacity.

To explore the effects of collagen XIII on lung mechanics, we measured the lung function of 3-month-old mice of both sexes with the flexiVent system. The lung volume, indicated by forced vital capacity (FVC), inspiratory capacity (IC) and an estimate of IC from the pressure-volume curves (A), was significantly increased in the *Col13a1^−/−^* mice relative to the wild-type, with the mean FVC 17% higher (figure 2A–C). In emphysema the peak flow parameters of NPFE are expected to show decreased values and increased length of expiration,^41^ but this was not the case in our measurements with forced expiratory volume in 0.1 s/FVC indicating that approximately 90% of the vital capacity was extracted during the first 0.1 s of exposure to negative pressure in all the genotypes (figure 2D). Tissue elasticity (H), which is measured using a different model, was similar in all the genotypes (figure 2E), whereas notable changes in H are reported in models of emphysema.^41^ Thus, we could not detect any signs of emphysema or bronchoconstriction behind the differences in lung volume. The sex distribution was such that the proportion of male mice was 33%–56%, and the changes were similar in both sexes when viewed separately. In addition, the weights of the mice were similar regardless of genotype, which excludes size differences as a cause (online supplemental figure 3). In the constant-phase model, static compliance (Cst) was increased, indicating that the extendibility of the lung and chest wall was increased in the *Col13a1^−/−^* mice (figure 2F). The *Col13a1^tm/tm^* mice did not show statistically significant differences relative to their wild-type littermates. The lung function measurements for the 3-month-old mice are presented in full in online supplemental figure 3. As expected, the lung volume increased from 3 months to 6 months of age, but by 6 months no statistically significant differences existed between the genotypes (online supplemental figure 4).

10.1136/bmjresp-2023-001850.supp3 Supplementary data

10.1136/bmjresp-2023-001850.supp4 Supplementary data

### Collagen XIII expression in pulmonary fibrosis

**Figure 3:**
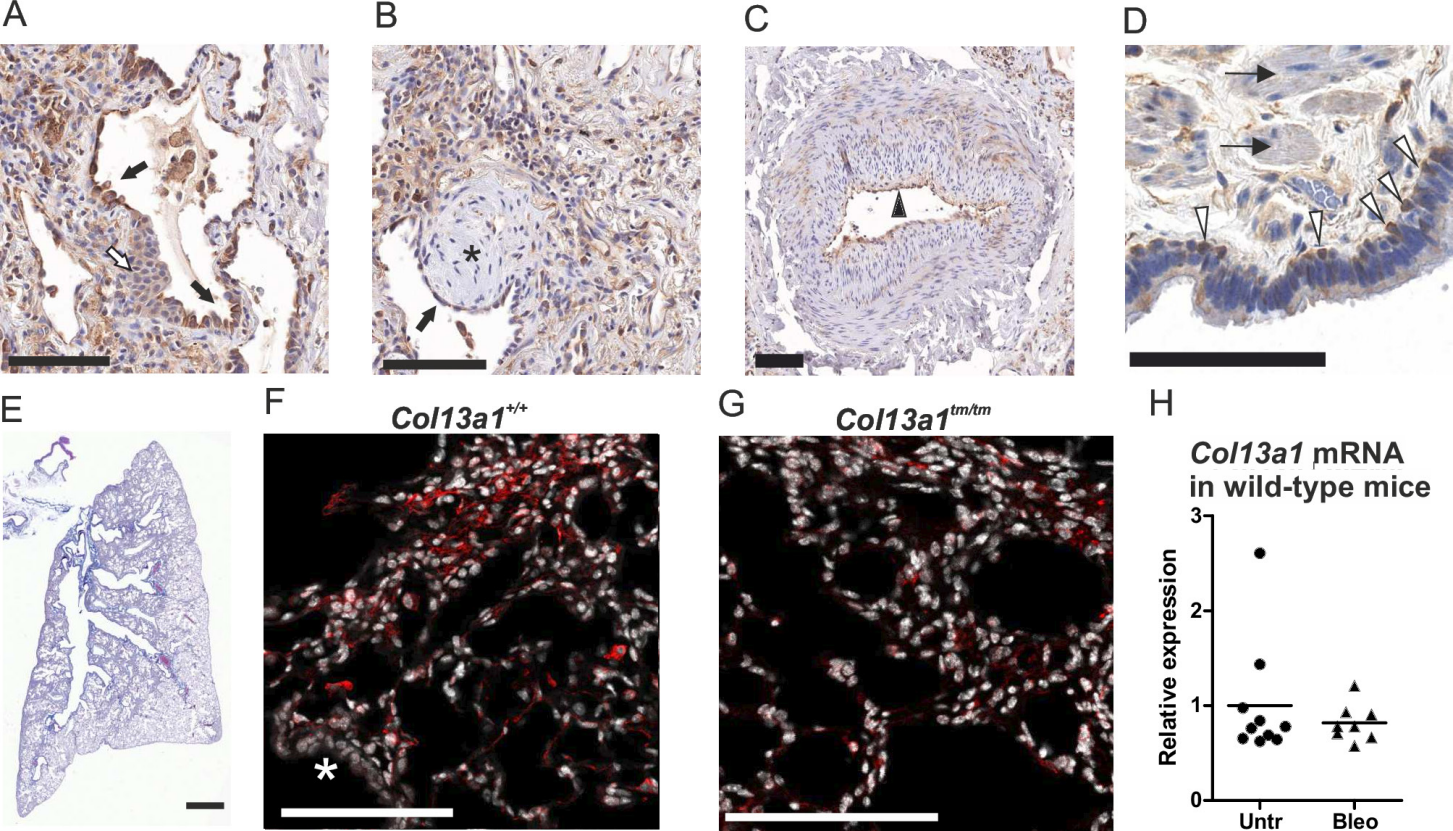
Collagen XIII expression in lung fibrosis. (A–D) Collagen XIII immunostaining of human IPF lungs. Black arrows: hyperplastic alveolar epithelium. White arrow: metaplastic squamous alveolar epithelium. Black asterisk: a fibroblast focus. Black arrowhead: Endothelium of an arteriole with a pathologically thickened wall. White arrowheads: bronchiolar basal cells. Thin black arrows: smooth muscle cells. Scalebars 100 µm. (E) Representative image of bleomycin-induced pulmonary fibrosis in wild-type mice. Scalebar 1 mm. Collagen XIII immunostaining (red) in (F) wild-type and (G) *Col13a1*^*tm/tm*^ mouse lung. Nuclei (DAPI) in white. White asterisk: bronchiole epithelium. Scalebars 100 µm. (H) *Col13a1* expression measured with RT-qPCR in wild-type untreated (Untr) and Bleomycin-treated (Bleo) mice. White balance and/or brightness have been adjusted for easier viewing. IPF, idiopathic pulmonary fibrosis.

To find out whether collagen XIII expression is altered in pulmonary fibrosis, we stained sections from human IPF lungs for collagen XIII. The fibrotic areas in the lungs exhibited stronger immunohistochemical expression for collagen XIII than the surrounding areas of nearly normal-looking lung tissue (figures 1A,B and 3A–D). The most intense collagen XIII expression was detected in the hyperplastic alveolar epithelium of the bronchiolised regions, while the adjacent squamous metaplasia showed low expression (figure 3A). Interstitial stromal cells in fibrotic regions were also broadly positive for collagen XIII (figure 3A,B), although the areas of dense fibrosis were less prominent (figure 3B). The fibroblastic foci were devoid of collagen XIII staining, but the cells lining them, which it is suggested may present aberrant basaloid cells,^28^ were positive for collagen XIII (figure 3B). An arteriole with pathological wall thickening gave an endothelial collagen XIII signal (figure 3C), and the basal cells of the bronchioles located in fibrotic areas also exhibited collagen XIII signals, while the smooth muscle cells were negative (figure 3D). Thus, we became interested in whether the altered collagen XIII expression would affect the development of pulmonary fibrosis and used the bleomycin experimental model to induce pulmonary fibrosis in wild-type, *Col13a1^−/−^* and *Col13a1*^*tm/tm*^ mice, the latter expressing only membrane-bound collagen XIII, with a 4-week follow-up for a strong histological effect (figure 3E). Collagen XIII expression was detected in the fibrotic lung regions of both wild-type (F) and *Col13a1^tm/tm^* (G) lungs, the latter exhibiting a somewhat restricted and punctuated staining pattern. In mice, we did not detect expression in morphologically epithelial-appearing cells. Despite the induced appearance of staining in the fibrotic regions, the overall *Col13a1* mRNA expression was not increased at 4 weeks postinduction (figure 3H).

### Lack of collagen XIII does not affect the development of pulmonary fibrosis

**Figure 4:**
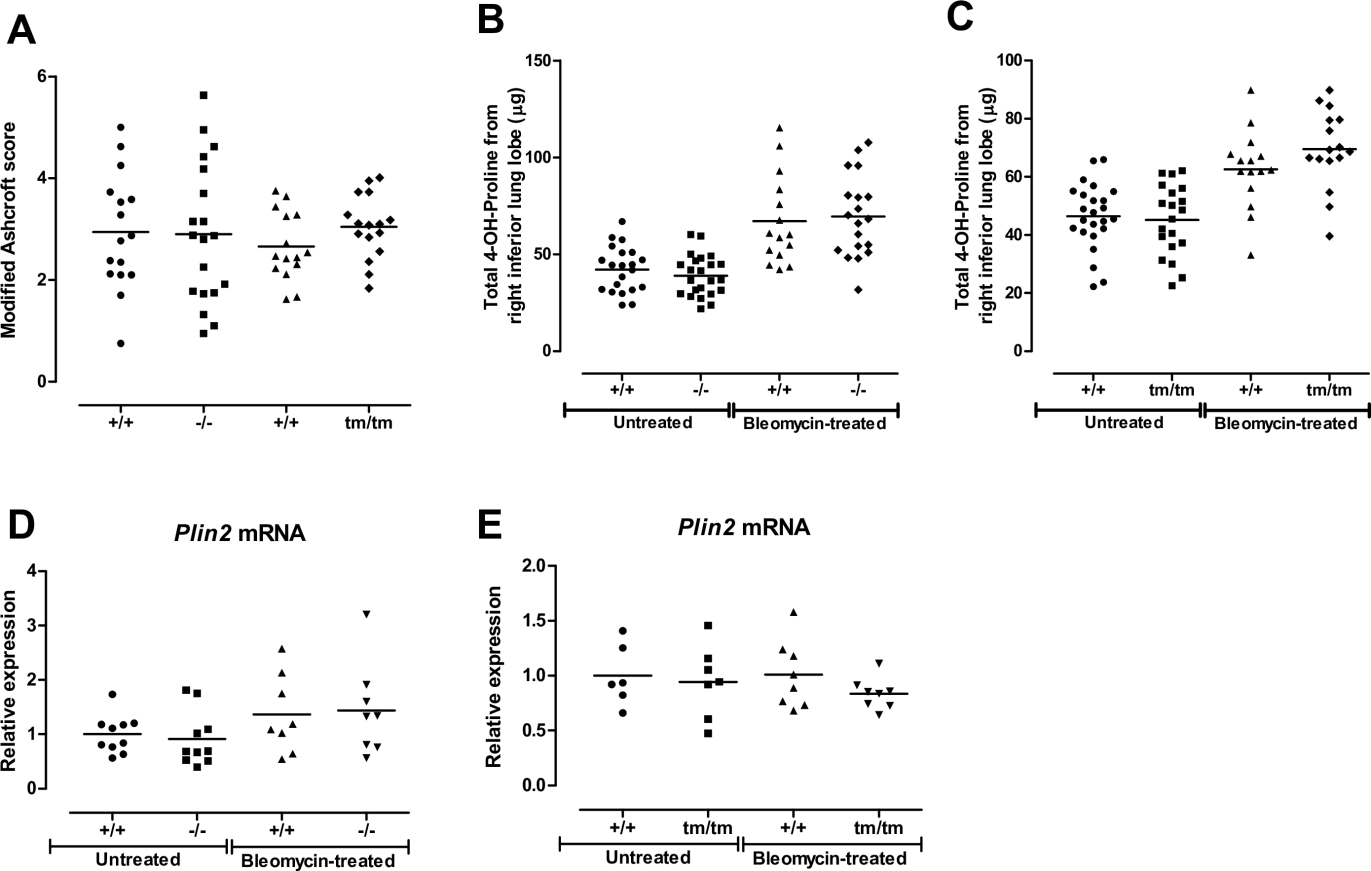
Development of fibrosis in *Col13a1*-modified mice at 4-week post bleomycin induction. (A) Histological scoring of fibrosis in the lungs of bleomycin-treated mice. N=15–19. (B, C) 4-Hydroxyproline measurements in untreated and bleomycin-treated *Col13a1*^*−/−*^ mice (−/−), *Col13a1*^*tm/tm*^ mice (tm/tm) and littermate wild-type mice (+/+). N=15–24.Expression of perilipin 2 mRNA in (D) *Col13a1*^*−/−*^ and (E) *Col13a1*^*tm/tm*^ mice measured with RT-qPCR. Lines at mean. N=6–10.

Since collagen XIII expression appeared to be induced in fibrotic areas, we proceeded to investigate whether mice with altered collagen XIII would be more susceptible to fibrotic changes. Induction of pulmonary fibrosis with bleomycin (figure 3E) revealed similar increases in the modified Ashcroft score (figure 4A) and 4-hydroxyproline content in *Col13a1*-modified mice to those in littermate wild-type controls (figure 4B,C). Lung function was measured in the bleomycin-treated mice, but no differences between the genotypes were observed (online supplemental figure 5). Thus, the larger lung volume of the knockout mice did not protect them from the restriction caused by pulmonary fibrosis. As the collagen XIII-positive cells are often classified as lipofibroblasts, we examined the levels of perilipin 2 (*Plin2*), a structural component of lipid droplets, which were similar in the different genotypes and treatments (figure 4D,E).

10.1136/bmjresp-2023-001850.supp5 Supplementary data

## Discussion

In 2010, we identified myasthenia in collagen XIII knockout mice^10^ and in further studies pointed out its importance for neuromuscular junction maturation, stabilisation and recovery postinjury.^19 42 43^ In 2015, loss-of function mutations in *COL13A1* were found in a novel disease subclass, CMS19,^4^ and to date altogether 19 causative mutations have been identified.^5–9 44^ Besides myasthenia, most CMS19 patients experience episodes of respiratory distress that require ventilation support,^5^ and a fatally progressing chronic lung disease contracted after multiple lung infections has also been reported in one case.^4^ Although collagen XIII expression in the lung is well established,^10 11^ its functional role in the lung and relevance to CMS19 pathophysiology have not been addressed. We demonstrate here with immunostainings that collagen XIII protein is located in the alveolar septal extracellular matrix, namely in basement membranes residing between the alveolar fibroblasts and type I or type II alveolar epithelial cells. The more restricted expression pattern in *Col13a1^tm/tm^* mice indicates that collagen XIII in the lung is at least partially shed into the pericellular matrix. In a bid to find out more about the role of collagen XIII, we first examined the structure of the *Col13a1^−/^*^−^ and *Col13a1^tm/tm^* mouse lungs by histology and electron microscopy, but no differences could be observed, indicating that the role of collagen XIII is functional rather than structural.

Most CMS19 patients exhibit restricted vital capacity,^5^ and collagen XIII knockout mice have been observed to breathe superficially.^19^ We, thus, questioned whether the lack of collagen XIII predisposes the individual to restrictive lung disease and studied the role of collagen XIII expression in lung fibrosis both in man and in the bleomycin-induced pulmonary fibrosis mouse model. We found out that collagen XIII is located in fibrotic areas of the lung in both IPF patients and bleomycin-treated mice. Interestingly, collagen XIII expression in the IPF lungs is induced in the basal epithelial cells and disease-committed hyperplastic alveolar epithelial cells, both possessing regenerative features and the latter also profibrotic ones. A collagen XIII signal was also observed in the endothelium of an arteriole which showed signs of pathology. Similarly, collagen XIII induction has been described earlier in renal endothelial cells in a mouse model for renal fibrosis and Alport’s syndrome.^45^ The vascular structures in the lung samples that were more normal in appearance were negative for the collagen XIII signal, suggesting that collagen XIII may be induced in endothelial pathology.

Despite being expressed in fibrotic areas, neither the lack of collagen XIII total protein nor the ectodomain affected the development of pulmonary fibrosis in the bleomycin model of pulmonary fibrosis. It must be remembered, however, that the single-dose bleomycin model does not express all the features of human IPF, just as bleomycin-induced pneumonitis in human patients presents typically with other histological findings rather than UIP.^46 47^ One of the major differences between IPF and the bleomycin model is the latter’s lack of fibroblastic foci and honeycombing which are hallmarks of IPF. Because the collagen XIII-positive hyperplastic epithelial cells are associated with these features, the mice are likely to lack these kinds of cells. As such, questions on the potential effects of collagen XIII deletion on these cells remain open.

The collagen XIII knockout mouse model recapitulates the human disease in many ways, one of these being superficial breathing.^19^ There were clear differences in lung capacity and extendibility in the collagen XIII knockout mice at 3 months, but no such differences could be observed any longer at 6 months. Considering that human CMS19 patients usually show a gradual improvement in their condition in adolescence, a similar effect cannot be ruled out in mice. In fact, the myasthenic phenotype is not progressive in adult mice.^43^ The techniques used for the lung function measurements differ from human lung function measurements in the regard that the mouse is paralysed, and the measurement does not reflect the activity of the respiratory muscles as opposed to human spirometry. Nevertheless, the techniques have been validated in different types of lung disease.

Both human patients and *Col13a1-*modified mice present with skeletal abnormalities^2 5^ and although not confirmed, thoracic changes cannot be ruled out in mice, though the changes in the human patients were mostly restrictive. Thus, we conclude that the differences found here are most likely due to increased extendibility of the thoracic cage as a result of the myasthenic phenotype. In human CMS19 patients, severe deformities of the thoracic spine such as kyphosis and scoliosis are observed, which are likely to limit their lung function. Although our findings did not assign any direct role for collagen XIII in the healthy lung, we were able to expose abnormalities in pulmonary properties in cases of collagen XIII deficiency. Furthermore, the disease-related induction of collagen XIII expression suggests its involvement in pulmonary remodelling processes, which may be relevant to pulmonary health in CMS19 patients. This warrants more in-depth studies on the significance of collagen XIII in lung physiology and pathology.

## Data Availability

Data are available on reasonable request. The data generated during this study are available from the corresponding author on reasonable request.
