# Supplementary material for: Contribution of collagen XIII to lung function and development of pulmonary fibrosis
Source: BMJ Open Respir Res. 2023 Dec 12;10(1):e001850. doi: 10.1136/bmjresp-2023-001850 (PMC10729248; doi:10.1136/bmjresp-2023-001850)
Supplement: Supplementary data [file bmjresp-2023-001850supp006.pdf]

Supplemental methods

Mice

The generation of *Col13a1* knockout (*Col13a1$^{-/-}$*) mice (B6.129-Col13a1$^{tm3.1Pih}$/Oulu; EM:09878; RRID:IMSR_EM:09878) is described in detail in (10). The genetic background of the mice originates from sv129 embryonic stem cells, followed by cross-breeding with the C57BL/6JOlaHsd strain for 17 generations and the C57BL/6NClr strain for 3-4 generations. The generation of *Col13a1$^{tm/tm}$* mice (B6JOlaHsd.129S6-Col13a1$^{tm4.1Pih}$/Oulu; EM09323; RRID:IMSR_EM:09323) is described in (29). Briefly, the furin cleavage site of collagen XIII was mutated from -RRRR- to -ATAA- to prevent cleavage of the membrane-bound collagen XIII by furin and furin-like proteases into soluble form. The genetic background of the mice originates from sv129 embryonic stem cells, followed by cross-breeding with the C57BL/6JOlaHsd strain for more than ten generations and the C57BL/6NClr strain for 6-9 generations. Wild-type littermates were used as controls. Both sexes were used in all the experiments. The mice were maintained in a specific pathogen-free (SPF) facility at +21 °C with a 12-hour light-dark cycle and were fed Teklad global 18% protein rodent diet with ad libitum access to food and water. The welfare of the mice was monitored daily. The mice were initially allocated to treatment groups so that half of each litter went into bleomycin treatment and for the rest lung function measurements were performed without bleomycin exposure. Randomization sequences were not applied. To minimize confounding, the mice were handled, treated, housed, and measured in the order of their identity numbers which are assigned at birth and are not connected to genotype or treatment group. Female littermates were housed in the same cage and male littermates were housed separately from the start of the experiments. A subset of the mice used here were also used in another publication on suitable quantitative PCR reference genes (33). The study protocols were prepared *a priori* and were not registered. The total number of mice was 233.

Lung function measurements

Three- and six months old mice were anesthetized with a subcutaneous injection of 0.4 mg/kg fentanyl, 10 mg/kg midazolam and 1.5 mg/kg medetomidine. The trachea was cannulated with an 18 G cannula from a

midline incision after local anesthesia with 0.1% lidocaine. The cannula was connected to a flexiVent FX Module 2 ventilator (SCIREQ, Montreal) equipped with a Forced Expiration Extension add-on. The mice were ventilated at 150 breaths per minute with a tidal volume of 10 ml/kg body weight and a positive end-expiratory pressure of 3 cmH_2O. Muscle relaxation was achieved with an intraperitoneal injection of 4 mg/kg body weight rocuronium bromide (B. Braun). Measurements were made initially on a pilot group of mice without rocuronium, using intermittent hyperventilation at 250 breaths per minute, but a large proportion of these mice exhibited spontaneous breathing efforts. Two recruitment maneuvers ("Deep Inflation") were run before the lung function measurements, followed by a broadband forced oscillation technique (FOT) ("QuickPrime-3") to confirm sufficient muscle relaxation. Lung function was measured by running a Deep Inflation maneuver, a single frequency FOT ("Snapshot-150"), a QuickPrime-3 maneuver, a stepwise pressure-volume loop ("PVs-P") and a negative pressure forced expiration ("NPFE") maneuver with approximately five-second intervals of regular ventilation between these measurements. Triplicate measurements were made on each mouse and averaged for the final values. Measurements with software-calculated coefficient of determination values below 0.95 for any maneuver and measurements that showed signs of airway instability, such as spontaneous breathing efforts, cannula obstruction or leaks, were excluded from the analysis.

The Deep Inflation recruitment maneuver inflates the lungs from end-expiratory volume to total lung capacity and measures the inspiratory capacity (IC). The Snapshot-150 is a 2.5 Hz single frequency FOT which measures volume, pressure and flow and fits them to a single compartment model and calculates total resistance (Rrs), elastance (Ers) and compliance (Crs). The QuickPrime-3 broadband FOT fits flow and pressure signals from different frequencies to the constant phase model to obtain Newtonian resistance (Rn), tissue damping (G) and tissue elastance (H). The PVs-P maneuver is a stepwise pressure-volume loop from PEEP to a pressure of 30 cmH_2O and back with volume and pressure signals fitted to the Salazar-Knowles equation to extract static compliance (Cst), an estimate of inspiratory capacity (A) and a shape constant (K). Finally, the NPFE maneuver inflates the lungs to total lung capacity and then exposes them to a negative pressure of -55 cmH_2O and measures the flow, volume and pressure changes to generate a flow-volume

curve for calculating parameters such as forced vital capacity (FVC), forced expiratory volume in 0.1 seconds (FEV0.1) and 0.2 seconds (FEV0.2), forced expiratory flow at 0.05 seconds (FEF0.05) and 0.1 seconds (FEF0.1) and peak expiratory flow (PEF).
