# Supplementary material for: Contribution of collagen XIII to lung function and development of pulmonary fibrosis
Source: BMJ Open Respir Res. 2023 Dec 12;10(1):e001850. doi: 10.1136/bmjresp-2023-001850 (PMC10729248; doi:10.1136/bmjresp-2023-001850)
Supplement: Supplementary data [file bmjresp-2023-001850supp001.pdf]

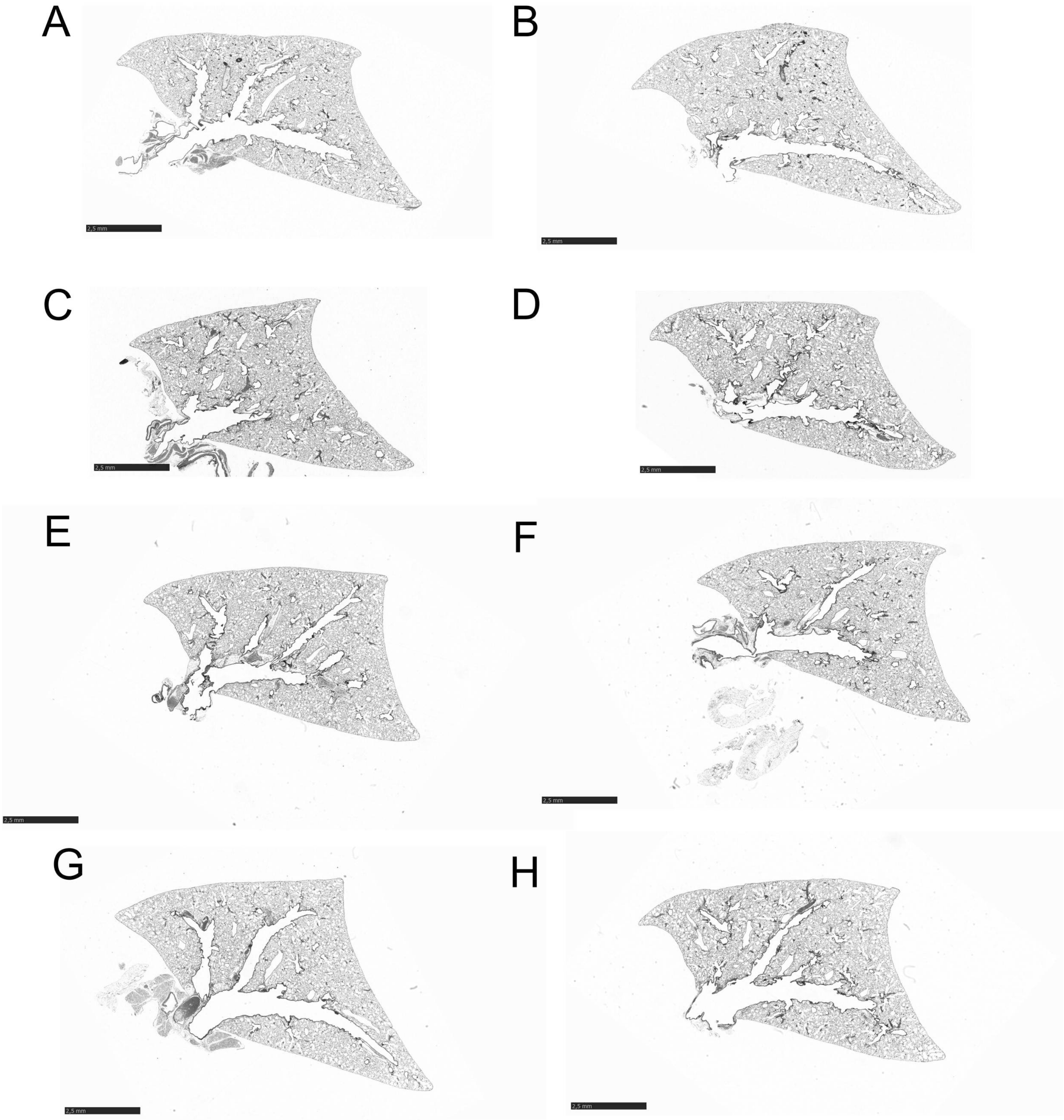

Supplemental figure 1. Lung histology of wild-type, *Col13a1*$^{-/-}$ and *Col13a1*$^{tm/tm}$ mice at given time points. Masson's trichrome staining of the left lung of three-month-old A) wild-type, B) *Col13a1*$^{-/-}$, C) wild-type and D) *Col13a1*$^{tm/tm}$ mice. Masson's trichrome staining of the left lung of six-month-old E) wild-type, F) *Col13a1*$^{-/-}$, G) wild-type and H) *Col13a1*$^{tm/tm}$ mice. Scalebars 2.5 mm.
