# Supplementary material for: Contribution of collagen XIII to lung function and development of pulmonary fibrosis
Source: BMJ Open Respir Res. 2023 Dec 12;10(1):e001850. doi: 10.1136/bmjresp-2023-001850 (PMC10729248; doi:10.1136/bmjresp-2023-001850)
Supplement: Supplementary data [file bmjresp-2023-001850supp002.pdf]

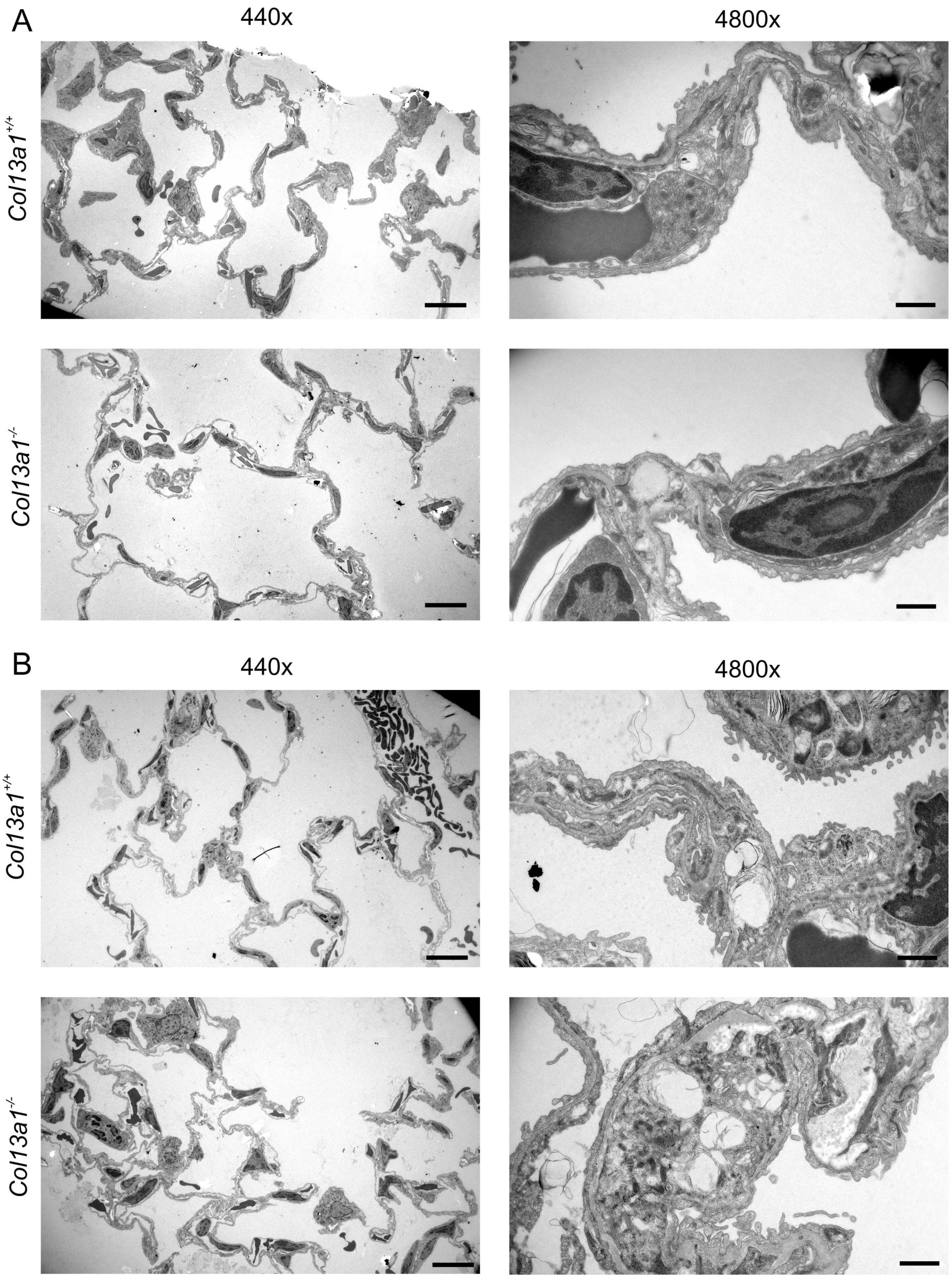

Supplemental figure 2. Representative TEM images of wild-type and *Col13a1*$^{-/-}$ mouse lungs at three months (A) and six months (B). Scalebars 10 µm in images with magnification 440x (left) and 1 µm with 4800x (right).
