# Supplementary material for: Contribution of collagen XIII to lung function and development of pulmonary fibrosis
Source: BMJ Open Respir Res. 2023 Dec 12;10(1):e001850. doi: 10.1136/bmjresp-2023-001850 (PMC10729248; doi:10.1136/bmjresp-2023-001850)
Supplement: Supplementary data [file bmjresp-2023-001850supp003.pdf]

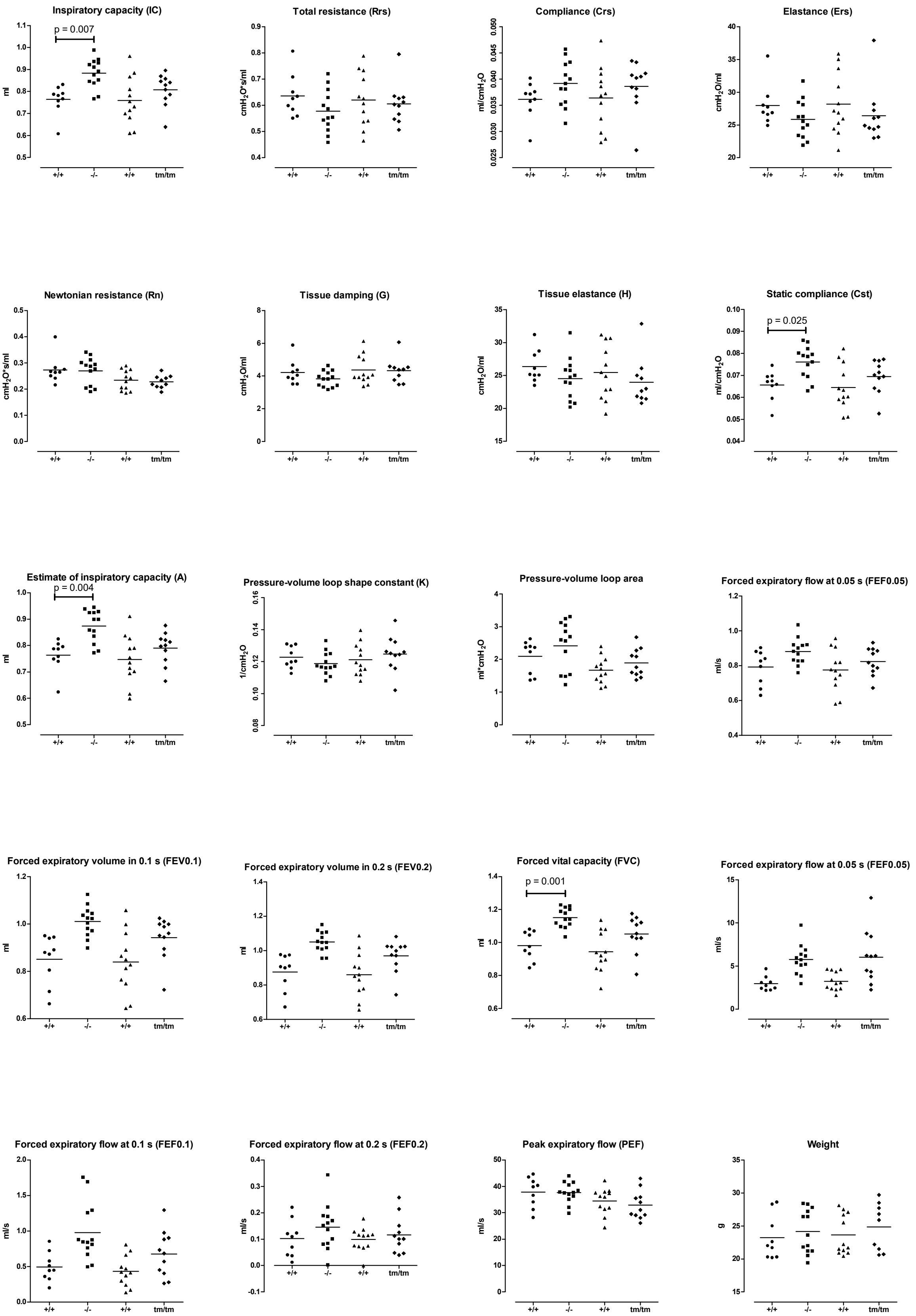

Supplemental figure 3. Lung function measurements of three-month-old untreated mice. +/+ = wild-type mice, -/- = *Col13a1*$^{-/-}$ mice. tm/tm = *Col13a1*$^{tm/tm}$ mice. Lines at mean.
